# Levamisole-induced Vasculitis in a Hepatitis C Patient: A General Medicine Ward Perspective on Diagnosis and Management

**DOI:** 10.7759/cureus.5198

**Published:** 2019-07-22

**Authors:** Zara Latif, Issa Pour-Ghaz, Jaclyn B Bergeron

**Affiliations:** 1 Internal Medicine, University of Tennessee Health Science Center, Memphis, USA; 2 Internal Medicine, Methodist University Hospital, Memphis, USA

**Keywords:** levamisole, vasculitis, cocaine, purpura, drug-induced, hepatitis c

## Abstract

Purpura in hepatitis C patients has a wide range of possible etiologies, some of which include mixed cryoglobulinemia and idiopathic thrombocytopenic purpura. Levamisole is a common chemical used as a lacing agent for cocaine. It is believed to enhance the addictive properties of cocaine, but it has been associated with a vasculitis syndrome that most commonly presents with purpura and leukopenia. We report a case of a patient with hepatitis C and cocaine abuse who presented with vasculitis, thrombocytopenia, and bloody sputum. A punch biopsy was performed, which confirmed the diagnosis of levamisole-induced vasculitis. A comprehensive rheumatology workup could result in variable serology and does not provide a definitive diagnosis. We suggest performing a punch biopsy as part of the initial workup for these patients, as it can provide rapid diagnosis and is associated with a lower cost.

## Introduction

Cocaine has been responsible for over 14,000 deaths in the United States in 2018 [1]. It has been estimated that 80% of the cocaine used in the United States is contaminated with levamisole. Levamisole is an anthelminthic agent that has been used as an antineoplastic drug and immunomodulator [2]. Its use was banned by the Food and Drug Administration (FDA) in 1999 due to reports of agranulocytosis and has been reported to be the culprit of cutaneous leukocytoclastic vasculitis [3-4]. In some cases, it has been shown to cause a vasculitis syndrome characterized by purpura, neutropenia, intravascular thrombosis, and pauci-immune crescentic glomerulonephritis in the presence of antineutrophil cytoplasmic antibodies (ANCA) [5].

The mechanism of action of levamisole is not well-understood, but it is hypothesized that it potentiates the dopaminergic effects of cocaine and enhances addiction [4]. Because of its short half-life and narrow window of detection in urine, widespread testing for levamisole levels is not used. Levamisole has a half-life of 5.6 hours and less than 5% is detected in urine testing [4]. While levamisole vasculitis syndrome is well-reported in the literature, the diagnostic workup and management of levamisole-induced vasculitis in hepatitis C patients are not well-reported.

We report a case of a patient with a known history of cocaine abuse and hepatitis C who presented with a rash and thrombocytopenia after recent cocaine abuse.

## Case presentation

A 56-year-old Caucasian male with a past medical history of chronic obstructive pulmonary disease, cocaine abuse, hepatitis C, and alcohol abuse presented to the emergency department with a three-day history of painful rash. The patient stated that the rash started after he was given a pill by a friend and described the rash as painful, but nonpruritic. He also endorsed constipation and abdominal pain and had a constant cough with some hemoptysis, which was present during the physical exam. The patient denied weight loss, fever, or diarrhea.

**Figure 1:**
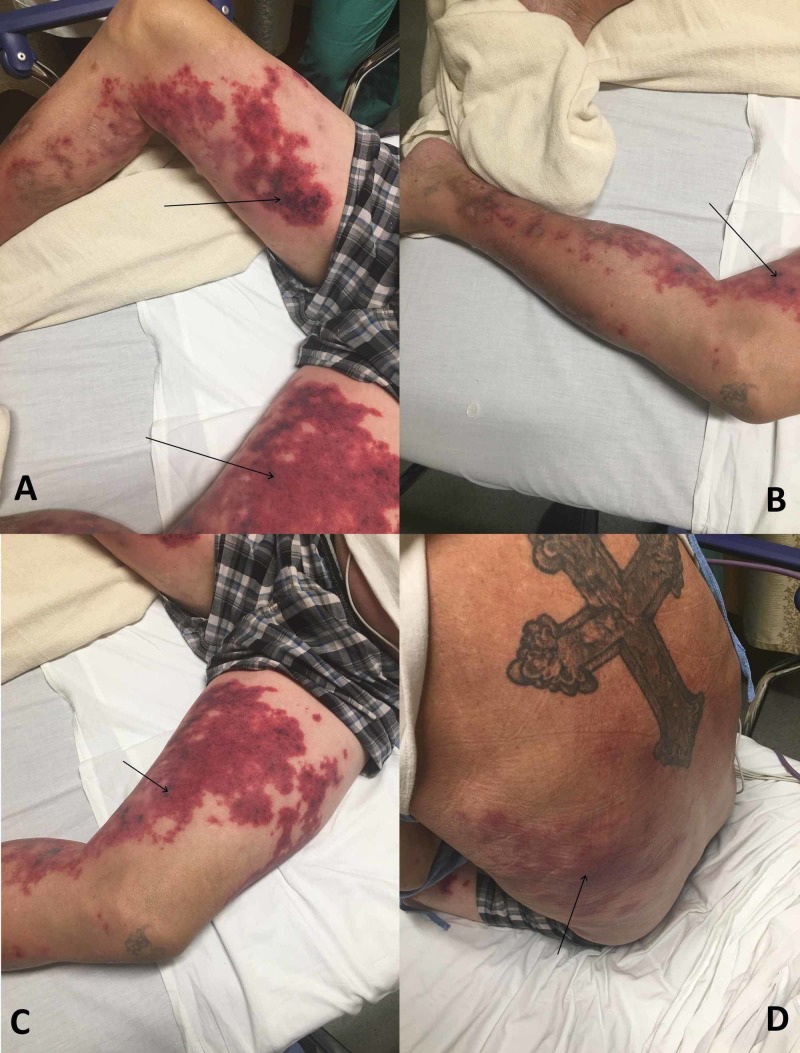
Levamisole-induced vasculitis in a cocaine user. Images obtained one week after cocaine use. Black arrows pointing to lesions scattered across his body shown in panels A, B, C, and D.

On presentation, the patient was afebrile, with a temperature of 36.9 degrees Celsius; tachycardic, with a heart rate of 100 beats per min; tachypneic, with a respiratory rate of 28 per minute; blood pressure was 108/76 mmHg; and oxygen saturation was 97% on room air. The physical exam was significant for coarse breath sounds bilaterally. He was coughing throughout the exam, with a small amount of bloody sputum production. He had bilateral scarlet-colored purpura that was contiguous and non-blanching (Figure 1). It covered his inner thighs and left flank. The purpura extended down both thighs, with deep purple lesions scattered throughout. Lesions were not raised, no skin breakdown could be appreciated, and some lesions were surrounded by hyperpigmented rings. He had mild erythema across the anterior abdomen and right flank.

**Figure 2:**
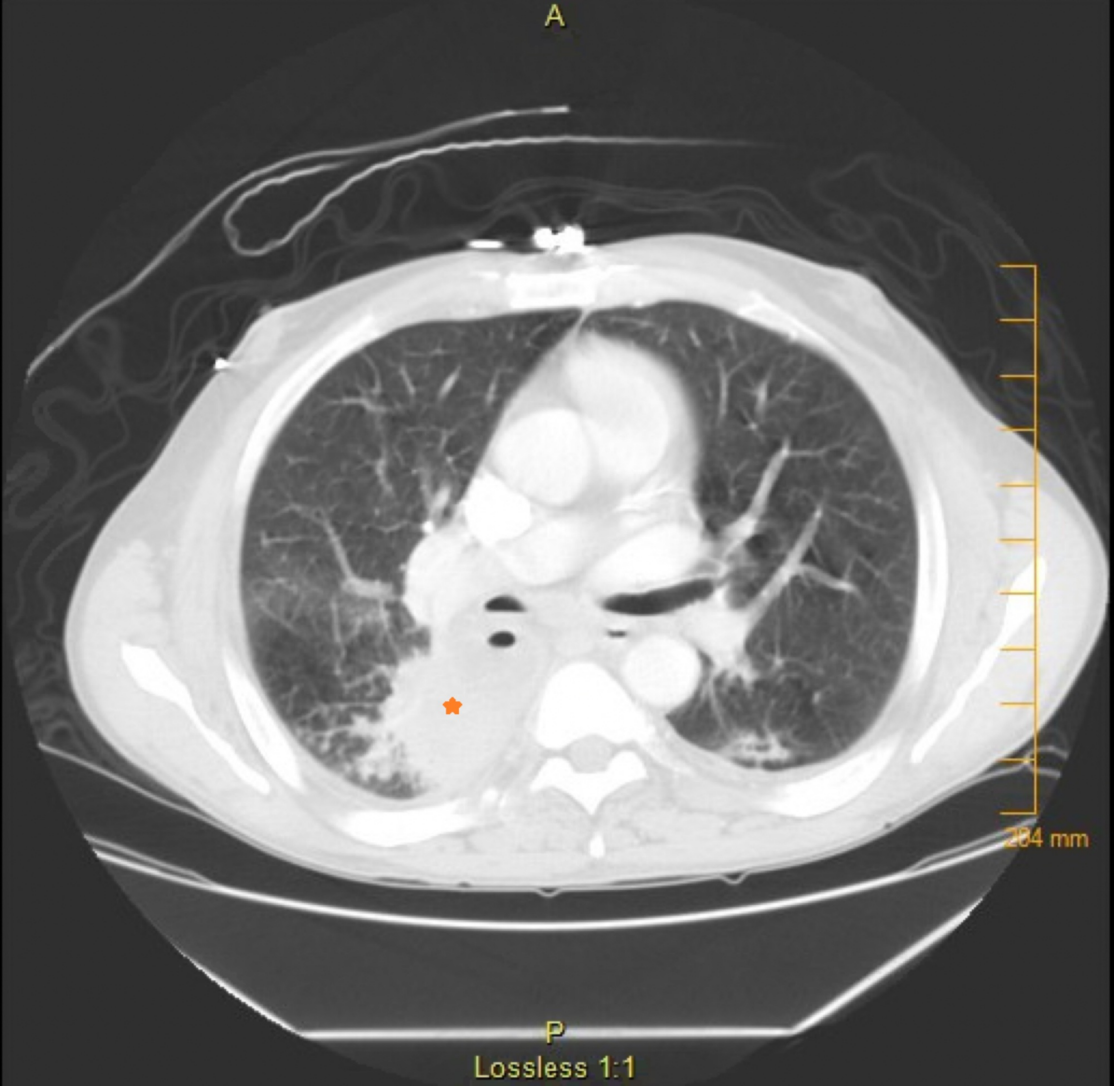
CT thorax with contrast showing a cavitary lesion in the right lower lung lobe. Orange star pointing to the consolidation. CT: computed tomography

**Figure 3:**
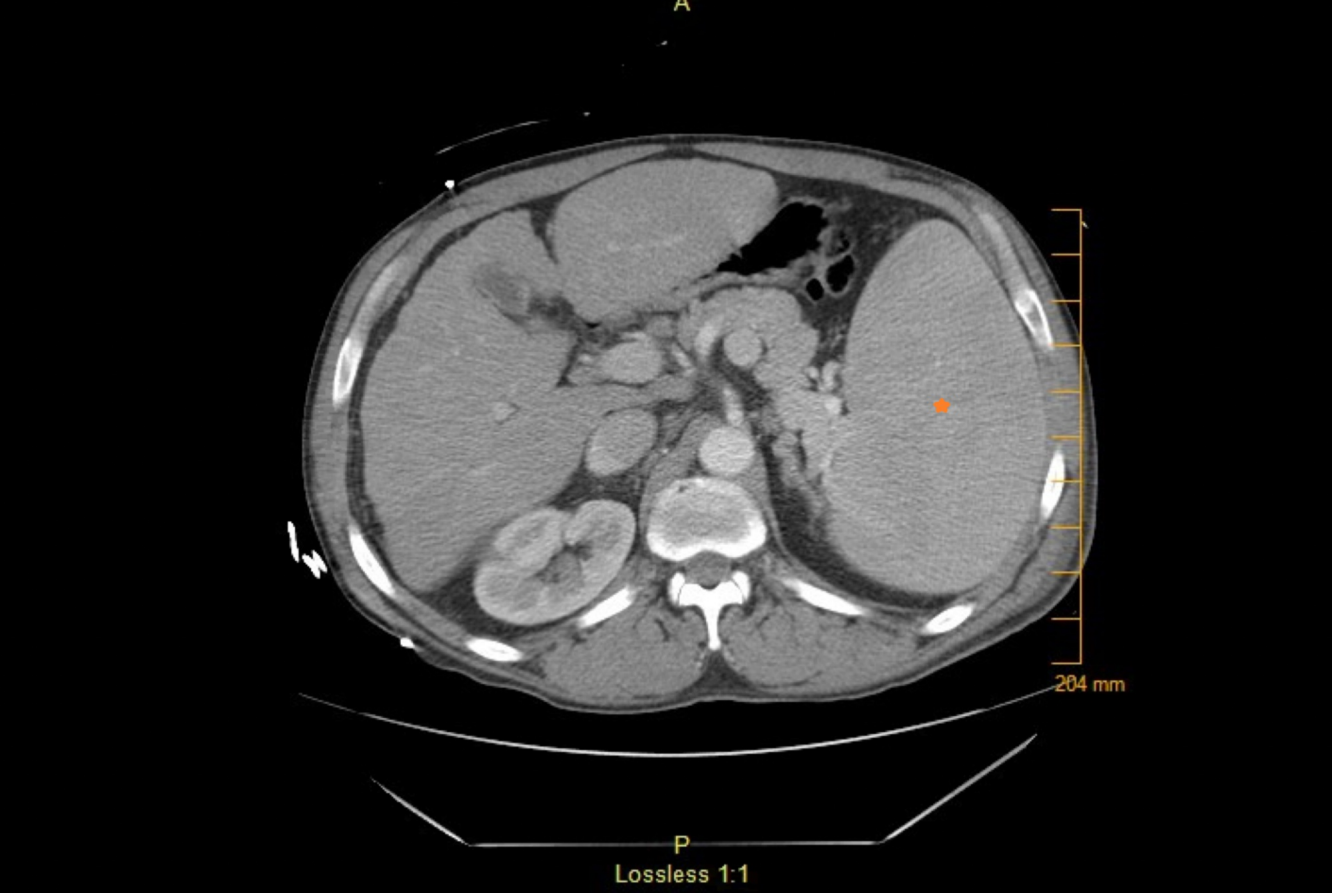
CT thorax with contrast capturing part of the abdominal cavity showing splenomegaly marked with the orange star. CT: computed tomography

CT thorax showed right lower lobe consolidation with an area of cavitation and possible necrosis (Figure 2). There were enlarged mediastinal lymph nodes, prominent axillary and retroperitoneal lymph nodes, cirrhosis, and splenomegaly (Figure 3). Lab findings were significant for leukopenia with a white blood cell (WBC) count of 2.6 (normal 4.2-10.2 thousand/mcL) and thrombocytopenia, with a platelet count of 106 (normal 150-400 thousand/mcL). The rest of the complete blood cell count values were within normal limits. Creatinine was 0.85 (normal 0.7-1.3 mg/dL) and remained normal during his hospitalization. Rheumatology workup showed an elevated rheumatoid factor (RF) at 19.9 (normal 0-15 IntUnits/mL ) and cryoglobulins were negative. C3 was low at 68.2 (normal 90-180 mg/dL), C4 was low at 6.2 (normal 10-40 mg/dL), anti-DNA double-stranded was normal at 9.7 (normal 0-24.9 IntUnits/mL), and anti-nuclear antibody was negative.

During the hospitalization, a punch biopsy of the lesions revealed occlusive thrombotic vasculopathy with an associated neutrophil-rich vasculitis affecting the small-caliber vessels. The findings were compatible with cocaine or levamisole-induced vasculitis. He was started on prednisone and Levaquin. After five days, the lesions resolved and the cough improved.

## Discussion

Purpura in hepatitis C patients with a history of cocaine abuse presents a unique diagnostic challenge due to the various possible etiologies of vasculitis. The differential diagnosis includes mixed cryoglobulinemia, idiopathic thrombocytopenic purpura (ITP), and levamisole-induced vasculitis. In this case, the patient also had hemoptysis, which further complicated his presentation due to concerns for possible malignancy. Levamisole-induced vasculitis could present as a syndrome with arthralgias, fever, leukopenia, neutropenia, and variable serology [6]. Skin lesions are often described as retiform purpuric patches along with cutaneous necrosis on the earlobes and nose [6]. Classic pathologic findings include leukocytoclastic vasculitis that involves small vessels with fibrinoid necrosis and a thrombotic pattern, including fibrin thrombi within the vessels [5]. Due to the highly suspicious presentation of these lesions, a punch biopsy was performed, and the patient was started on treatment for community-acquired pneumonia and was given steroids for the skin lesions.

Mixed cryoglobulinemia (CG) could be a cause of this patient’s vasculitis due to the history of hepatitis C. Mixed CG syndrome presents with arthralgia, weakness, and purpura, which is also known as Meltzer’s triad [7]. While our patient did have purpura and arthralgia, his biopsy results did not correspond with that of mixed CG and his cryoglobulin screen was negative. Biopsy in mixed CG usually shows the deposition of circulating immune complexes in small vessels [7]. Additionally, mixed CG has multi-organ involvement, including the kidneys, peripheral nerves, skin, and salivary glands. Mixed CG patients usually present with high RF levels and reduced C4 levels. The only test that can help definitively differentiate these two diseases is a biopsy. While levamisole-induced vasculitis can be observed without steroid treatment, mixed CG that is severe is treated with a steroid course or with rituximab when it is refractory to treatment, followed by anti-HCV treatment [8]. ITP was another possible etiology for this patient’s vasculitis [9]. It is a unique cause of vasculitis in hepatitis C patients, as there is an increased prevalence of ITP in hepatitis C patients [10]. Here, thrombocytopenia and palpable purpura could support this diagnosis, but the biopsy results would differ.

Due to the history and presentation, levamisole-induced vasculitis was the most likely diagnosis in this patient. He had a history of cocaine abuse, and cocaine was present in his urine. On laboratory workup, C3 and C4 were low and the punch biopsy results showed occlusive thrombotic vasculopathy. While our patient had lesions only on his thighs and torso, the lesions had a similar appearance to that described in the literature. The workup of these patients could be focused and a complete rheumatology panel is not necessary for establishing a diagnosis. Obtaining cryoglobulin levels, C3, C4, and anti-neutrophil cytoplasmic antibody could be helpful but will not provide a definitive diagnosis. We propose that the best way to establish this diagnosis is with a punch biopsy. A punch biopsy is a simple bedside procedure that could be performed on a general medicine service by using simple tools. Refraining from ordering an extensive rheumatology panel and eliminating unnecessary consults can help provide more streamlined patient care and shorten hospital stay for those patients.

The biopsy results in patients presenting with purpura is of immense importance. Due to increasing cocaine use in our society, a urine drug screen and biopsy of the lesion should be part of the initial workup for every patient presenting with purpura when the common causes are excluded and a cause cannot be identified. History and physical exam findings will often guide the diagnostic workup; however, this diagnosis is often challenging due to a reluctance to openly disclose illicit drug abuse. In our patient, his history of hepatitis C and cocaine use made levamisole-induced vasculitis the most likely cause, however, his presentation also raised concerns of malignancy and possibly an autoimmune process that could be responsible for his vasculitis. Due to the variability in serology, a punch biopsy was an invaluable tool to help confirm the diagnosis.

## Conclusions

Purpura in patients with a history of hepatitis C and cocaine abuse can present a diagnostic dilemma. With the increased cocaine use in our society, the possibility of levamisole-induced vasculitis should always be considered in the diagnostic workup. We suggest performing a punch biopsy as part of the initial workup for hepatitis C patients presenting with purpura. Due to the simplicity and low complication rates of this procedure, it as an ideal choice when evaluating these patients. We also recommend refraining from ordering a comprehensive rheumatologic panel, as serology could be variable and often nonspecific. Rheumatology can be a valuable tool if clinical suspicion is high for another pathologic process and the best clinical judgment should be applied to each case. This approach could significantly shorten the length of hospital stay, limit unnecessary costs, and improve patient care.
